# Enhanced clustering-based differential expression analysis method for RNA-seq data

**DOI:** 10.1016/j.mex.2023.102518

**Published:** 2023-12-12

**Authors:** Manon Makino, Kentaro Shimizu, Koji Kadota

**Affiliations:** a Graduate School of Agricultural and Life Sciences, The University of Tokyo, Yayoi 1-1-1, Bunkyo-ku, Tokyo 113-8657, Japan; b Interfaculty Initiative in Information Studies, The University of Tokyo, Hongo 7-3-1, Bunkyo-ku, Tokyo 113-0033, Japan; c Collaborative Research Institute for Innovative Microbiology, The University of Tokyo, Yayoi 1-1-1, Bunkyo-ku, Tokyo 113-8657, Japan

**Keywords:** RNA sequence (RNA-seq), Differentially expressed gene (DEG), Model-based clustering, R package, MBCdeg3

## Abstract

RNA-seq is a tool for measuring gene expression and is commonly used to identify differentially expressed genes (DEGs). Gene clustering has been widely used to classify DEGs with similar expression patterns, but rarely used to identify DEGs themselves. We recently reported that the clustering-based method (called MBCdeg1 and 2) for identifying DEGs has great potential. However, these methods left room for improvement. This study reports on the improvement (named MBCdeg3). We compared a total of six competing methods: three conventional R packages (edgeR, DESeq2, and TCC) and three versions of MBCdeg (i.e., MBCdeg1, 2, and 3) corresponding to three different normalization algorithms. As MBCdeg3 performs well in many simulation scenarios of RNA-seq count data, MBCdeg3 replaces MBCdeg1 and 2 in our previous report.

• MBCdeg3 is a method for both identification and classification of DEGs from RNA-seq count data.
• MBCdeg3 is available as a function of R, which is common in the field of expression analysis.
• MBCdeg3 performs well in a variety of simulation scenarios for RNA-seq count data.

## Graphical abstract

**Figure:**
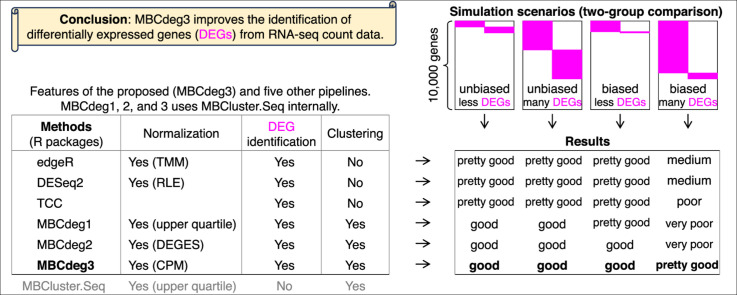

Specifications table

**Table:** 

Subject area:	Bioinformatics
More specific subject area:	Gene expression analysis
Name of your method:	MBCdeg3
Name and reference of original method:	MBCdeg1 and 2: T. Osabe, K. Shimizu, K. Kadota, Differential expression analysis using a model-based gene clustering algorithm for RNA-seq data, BMC Bioinformatics, 22 (2021) 511. https://doi.org/10.1186/s12859-021-04438-4 MBCluster.Seq: Y. Si, P. Liu, P. Li, T.P. Brutnell, Model-based clustering for RNA-seq data. Bioinformatics 30 (2014) 197-205. https://doi.org/10.1093/bioinformatics/btt632
Resource availability:	MBCdeg's original GitHub site https://github.com/takosa/MBCdeg-paper/ (last access: October 23th, 2023)

## Method details

### Background

One of the most common reasons for analyzing RNA-seq data is to identify DEGs for different groups or conditions [1], [2], [3]. Many studies have been reported on efficient and accurate means of identifying DEGs (e.g., [4,5]). Conventional differential expression (DE) analysis such as edgeR [6] and DESeq2 [7] typically consists of two steps (data normalization and DEG identification), and each R package has its own methods for each step [8]. These methods typically take as input a numerical matrix (called “count data”) whose rows are genes and columns are samples, and return *p*- and/or *q*-values assigned to individual genes. Researchers rely on these values to rank genes by degree of DE and consider a subset of genes that meet the false discovery rate (FDR) threshold as DEGs.

Following the identification of DEGs, gene clustering has occasionally been used (e.g., [9]). For example, the R package MBCluster.Seq [10] takes the reduced matrix of DEGs with a preselected number of clusters *K* and returns representative expression patterns for individual clusters and cluster numbers for individual genes. The DE analysis can be considered as a form of gene clustering [11]. For example, if one applies *K*-means clustering with *K* = 3 to count data comparing two groups (G1 vs. G2), one would expect the resulting expression patterns of the three cluster centers to be (i) up-regulated in G1 (“DEG1” pattern), (ii) up-regulated in G2 (“DEG2”), and consistent in both groups (“non-DEG”). The lower the posterior probability (PP) assigned to the “non-DEG” cluster, the higher the degree of DE for that gene [12]. Therefore, in principle, clustering-based DE analysis methods perform DEG identification and gene clustering simultaneously.

We have recently proposed a clustering-based DE pipeline (called “MBCdeg”) that implements this idea with MBCluster.Seq, and evaluated two versions of the pipeline, denoted as MBCdeg1 and MBCdeg2, with different data normalization methods [13]. MBCdeg1 uses the default normalization algorithm implemented in MBCluster.Seq, which adjusts the upper quartile of the counts of all genes between samples. MBCdeg2 uses the DEGES normalization algorithm [14] in TCC [15], which adjusts the trimmed mean of the counts of potential non-DEGs. Recently, an increasing number of papers have been evaluated using extensive simulation scenarios in which the proportion of DEGs (*P_DEG_*) in the data exceeds 50% (i.e., *P_DEG_* > 50%) [16], [17], [18], and such real-world data do exist [19]. Although MBCdeg1 and 2 generally perform significantly worse at *P_DEG_* > 50% [13], the basic counts per million (CPM) normalization method may perform relatively well under these conditions [16]. Here, we present an improved pipeline with CPM in the MBCdeg algorithm (named MBCdeg3).

### Algorithm

The concept of identifying DEGs based on gene clustering is described in Osabe et al. [13], and the pipeline is based on the R functions of MBCluster.Seq [10]. The only difference between the proposed pipeline (MBCdeg3) and the other MBCdegs (i.e., MBCdeg1 and 2) is the normalization step. Therefore, for consistency and clarity, we have used notations that were similar to those described in the original papers [10,13]. We denote an input count matrix as one where each row represents a gene *g* (= 1, …, *G*), each column represents a replicate *j* (= 1, …, *n_i_*) of group *i* (= 1, …, *I*), and each cell represents the number of counts. Where *G* is the number of genes, *I* is the number of groups compared, and *n_i_* is the number of replicates for the group *i*.

MBCluster.Seq clusters gene vectors *β_g_* = (*β_g_*_1_, …, *β_gI_*), where *β_gi_* indicates the count of gene *g* in the group *i* relative to the overall mean on a log-scale. Therefore, the sum of *β_gi_* for a gene *g* across all compared groups will be 0. *β_g_* can be considered as the log fold-change (*FC*) between compared groups. Given a preselected number of clusters *K*, MBCluster.Seq returns two results as output. One is the center for cluster *k, μ_k_* = (*μ_k_*_1_, …, *μ_kI_*) for *k* = 1, …, *K*, and the other is the PP that gene *g* belongs to the *k*^th^ cluster, *p_g_* = (*p_g_*_1_, …, *p_gK_*) for *g* = 1, …, *G*. To identify the non-DEG cluster, MBCdeg considers the *L*^2^ Norm of *μ_k_* for each cluster center across groups, ||*μ_k_*||_2_ = (|*μ_k_*_1_|^2^ + … + |*μ_kI_*|^2^)^1/2^. Since a smaller value of the norm for cluster *k* indicates a lower degree of DE across groups for that cluster, one can consider the probability of a gene being located in the *k*^th^ column as being in the non-DEG cluster, where *k* = argmin(||*μ*_1_||_2_, …, ||*μ_K_*||_2_). A lower value of the PP for gene *g* in the *k*^th^ cluster (i.e., *p_gk_*) indicates a higher degree of DE between the compared groups.

**Fig. 1:**
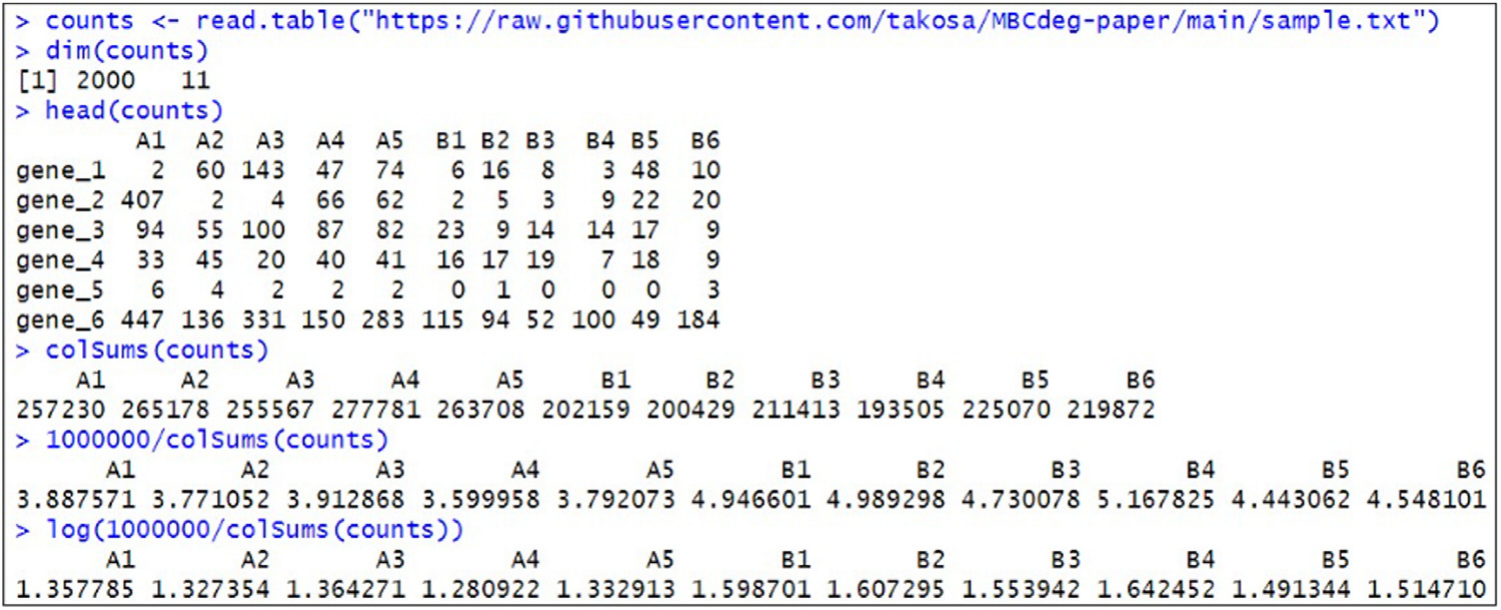
Screenshot of the R console for obtaining CPM normalization factors.

MBCluster.Seq consists of three functions (*RNASeq.Data, KmeansPlus.RNASeq*, and *Cluster.RNASeq*) that are designed to be used in sequential order. The *Normalizer* option in the *RNASeq.Data* function corresponds to data normalization. For MBCdeg3, the option specifies the logarithm of the CPM normalization factors. Fig. 1 is an example of the calculation of “log(CPM normalization factors)” using a simulation count dataset provided on Osabe's GitHub page (https://raw.githubusercontent.com/takosa/MBCdeg-paper/main/sample.txt). This dataset consists of 2,000 genes × 11 samples, comparing five Group A and six Group B samples (labeled “A1”, …, “A5”, “B1”, …, “B6”), where *G* = 2,000, *I* = 2, *n_A_* = 5, and *n_B_* = 6. The CPM is the number of counts of a gene in a sample converted to a value if the total counts in the sample were one million. For example, the total counts for sample “A1” were 257,230. Therefore, the normalization factor to obtain the CPM value can be obtained as 1,000,000/257,230 = 3.887571 and the logarithm is 1.357785.

**Fig. 2:**
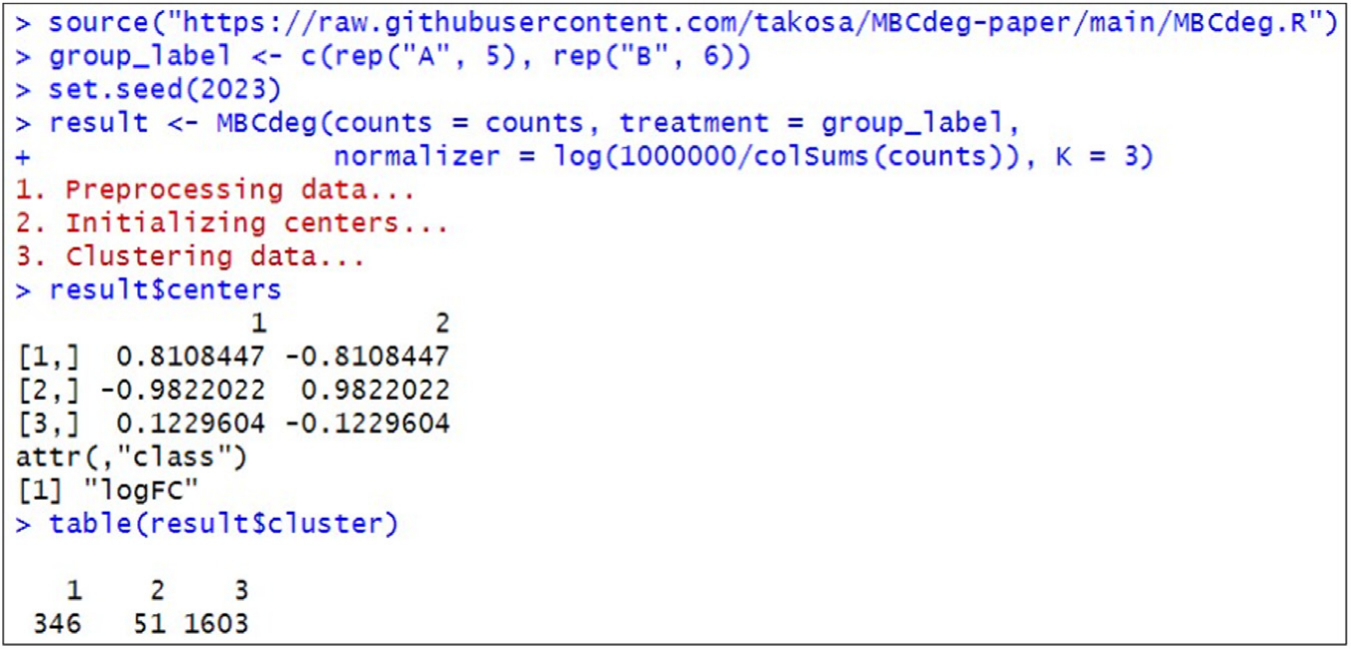
Screenshot of the results using the MBCdeg3.

Fig. 2 is an example of the execution for MBCdeg3 with the sample data. We used a wrapper function (called “*MBCdeg*”) provided on Osabe's GitHub page (https://raw.githubusercontent.com/takosa/MBCdeg-paper/main/MBCdeg.R). It takes a total of four arguments: raw count data, group label information, log-transformed CPM normalization factors, and the preselected number of clusters, *K*. For this sample data, MBCdeg3 with *K* = 3 returns the centers for cluster *k* (=1, 2, 3) as *μ*_1_ = (0.81, -0.81) with 346 genes, *μ*_2_ = (-0.98, 0.98) with 51 genes, and *μ*_3_ = (0.12, -0.12) with 1,603 genes. These results basically indicate that *μ*_1_ and *μ*_3_ have the DEG1 pattern and *μ*_2_ has the DEG2 pattern. In this case, the third cluster with the smallest norm (||*μ*_3_||_2_ = 0.174) is detected as the non-DEG cluster and the PP values are used for gene ranking.

### Method validation

**Fig. 3:**
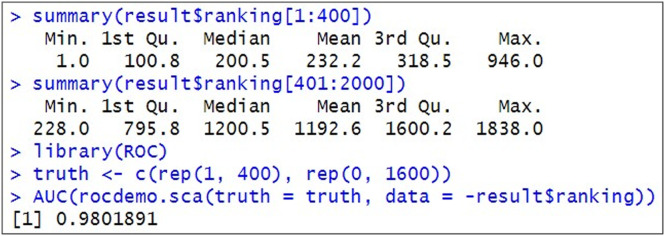
Screenshot of the distribution of gene ranking results and AUC value.

This sample data consists of the first 360 genes (gene_1, gene_2, ..., and gene_360) with the DEG1 pattern (up-regulated 4-fold in Group A), the next 40 genes (gene_361, gene_362, ..., and gene_400) with the DEG2 pattern (up-regulated 9-fold in Group B), and the remaining 1,600 genes (gene_401, gene_402, ..., gene_2000) with the non-DEG pattern. Therefore, the simulation scenarios are *P_DEG_* = (360 + 40)/2,000 = 0.2, the proportion of genes up-regulated in Group A (*P*_1_) = 360/(360 + 40) = 0.9. The DEG1 pattern up-regulated in 4-fold in A means that the ideal *μ*_1_ is (0.69, -0.69), with log_e_(4^1/2^) = 0.69. Similarly, the DEG2 pattern up-regulated in 9-fold in B means that the ideal *μ*_2_ is (-1.1, 1.1), with log_e_(9^1/2^) = 1.1 and the non-DEG pattern (up-regulated in 1-fold in both groups) means that the ideal *μ*_3_ is (0, 0), with log_e_(1^1/2^) = 0. The actual results obtained with MBCdeg3 shown in Fig. 2 are quite close to the ideal results. We confirmed that the area under the receiver operating characteristic curve (AUC) calculated using the ranked gene list and the corresponding list of true DEGs or non-DEGs was close to 1 (Fig. 3).

The current study is positioned to improve our MBCdeg's algorithm [13], which compared a total of five DE methods/packages (edgeR, DESeq2, TCC, MBCdeg1, and MBCdeg2), primarily using simulated data generated by the *simulateReadCounts* function in TCC. Following the previous study, we demonstrate the performance of MBCdeg3 as well as the above five methods based on the same evaluation metric and simulation framework. We use the AUC value as the comparison metric, which evaluates both sensitivity and specificity of the methods simultaneously. The simulation conditions are as follows: (i) the total number of genes is 10,000 (*G* = 10,000), (ii) two-group comparison (G1 vs. G2) with the same number of replicates per group (*n*_1_ = *n*_2_ = 3), (iii) the levels of DE are 4-fold in each group (*FC* = 4), (iv) 5, 25, 45, and 65% of the genes are DEGs (*P_DEG_* = {0.05, 0.25, 0.45, 0.65}), and (v) 50, 70, 90, and 100% of the DEGs are up-regulated in G1 (*P*_1_ = {0.5, 0.7, 0.9, 1.0}). A higher *P*_1_ value indicates a higher degree of up-regulated DEGs in G1, ranging from unbiased (*P*_1_ = 0.5) to completely biased (*P*_1_ = 1.0) conditions.

**Fig. 4:**
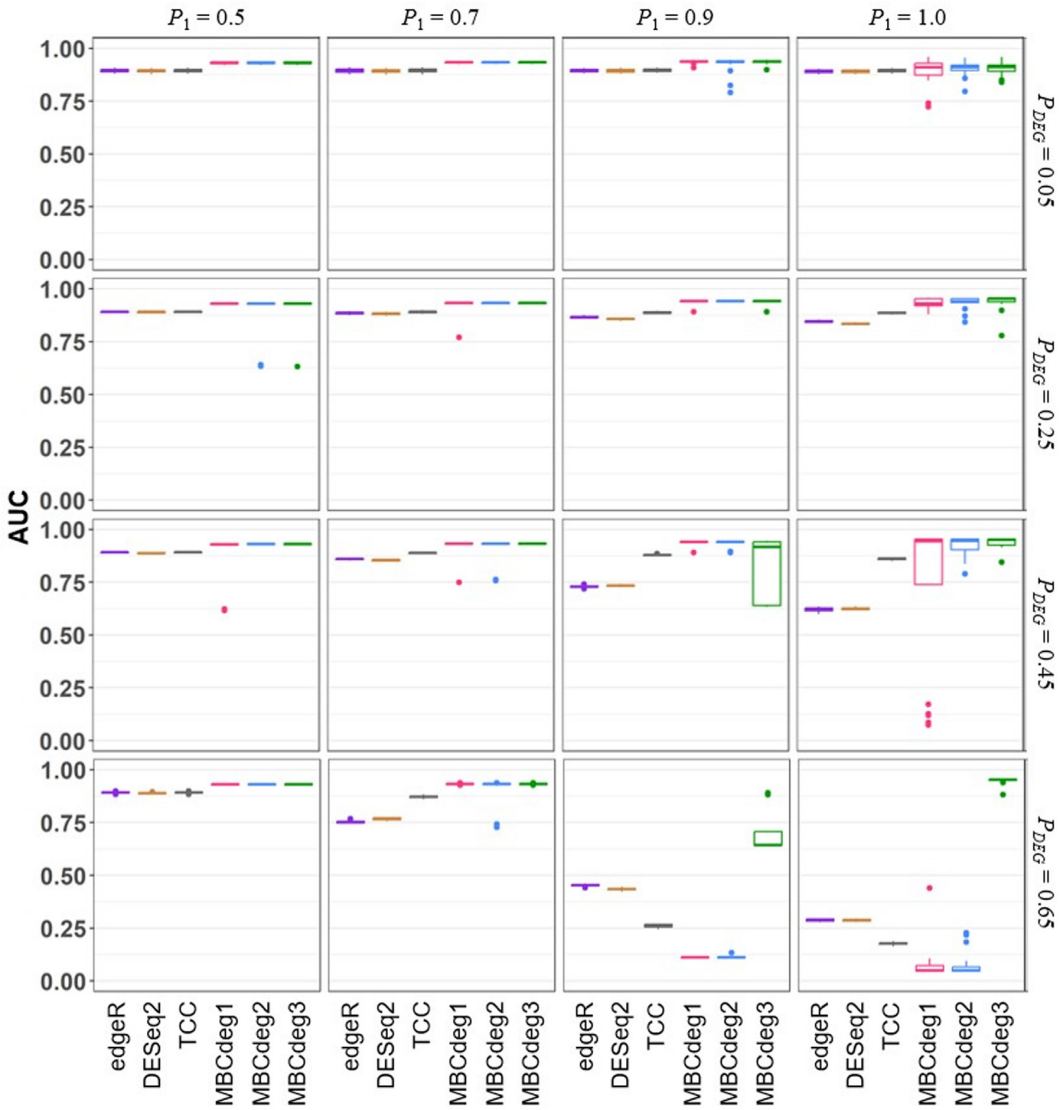
Simulation results.

Fig. 4 shows the AUC values for the six methods using a total of 16 simulation conditions, with 20 trials per condition. Note that the values except for MBCdeg3 are essentially the same as those in our previous study (i.e., Figs. 1 and 3 of [13]). Overall MBCdeg3 outperformed the others especially in the biased conditions (*P*_1_ > 0.5). There are two possible reasons for this. First, the CPM normalization method used in MBCdeg3 performs well at high *P_DEG_* and *P*_1_ values (e.g., *P_DEG_* = 0.65 and *P*_1_ = 0.9), but rather poorly at low *P_DEG_* values [16]. Second, the MBCdeg algorithm is robust at relatively low *P_DEG_* values (< 0.5), regardless of the choice of normalization methods, where the non-DEG cluster can be correctly identified in most cases [13].

Boxplots of the AUC values (20 trials) for each method under a total of 16 conditions, *P*_1_ = 0.5 (left) to 1.0 (right) with *P_DEG_* = 0.05 (top) to 0.65 (bottom), are shown.

Note that MBCdeg3 is clearly inferior to MBCdeg1 and 2 at *P_DEG_* = 0.45 and *P*_1_ = 0.9. Under this condition, we observed 13 trials with AUC ≈ 0.91 and 7 trials with AUC ≈ 0.64 for MBCdeg3. As discussed previously [13], most of the low AUC values are mainly due to misidentification of the non-DEG cluster. Since the number of DEG2 patterns in this condition is extremely small (= 450) compared to the others (4,050 for DEG1 and 5,500 for non-DEG), the cluster containing many DEG2 patterns is relatively unstable and leads to misidentification. The lowest average AUC value for MBCdeg3 of all conditions was found at *P_DEG_* = 0.65 and *P*_1_ = 0.9, where we observed 5 trials with AUC ≈ 0.89 and 15 trials with AUC ≈ 0.64. The low AUC values in this condition can also be explained by the above reason. Overall, MBCdeg3 shows a gradual decrease in performance as *P_DEG_* goes from 0.25 to 0.65 at *P*_1_ = 0.9. The fact that MBCdeg3 did not show AUC values lower than 0.63 in all conditions examined here may be an effect of CPM normalization. To our knowledge, there is no other method that can successfully rank genes in a wide range of simulation scenarios, from relatively easy conditions (e.g., *P_DEG_* = 0.05 and *P*_1_ = 0.5) to inherently difficult conditions (*P_DEG_* = 0.65 and *P*_1_ = 1.0). Therefore, the outstanding performance of MBCdeg3 demonstrated in this study is worth reporting.

## Conclusion

MBCdeg has the unique ability to perform pattern classification by gene clustering in a single step, which is not possible with traditional methods. Since the potential of MBCdeg3 is remarkable, we recommend trying it instead of MBCdeg1 or 2.

## Ethics statements

None.

## CRediT author statement

**Manon Makino:** Conceptualization, Methodology, Visualization, Investigation, Writing. **Kentaro Shimizu**: Methodology, Supervision, Validation, Reviewing. **Koji Kadota**: Methodology, Investigation, Visualization, Confirmation, Writing- Reviewing and Editing.

## Declaration of interests

The authors declare that they have no known competing financial interests or personal relationships that could have appeared to influence the work reported in this paper.

## Data Availability

The data and codes are available on Supplementary materials.

## Appendix Supplementary materials

Supplementary material All analyses were performed using R (ver. 4.2.3). The versions of the R packages used were edgeR ver. 3.40.2, DESeq2 ver. 1.38.3, TCC ver. 1.38.0, MBCluster.Seq ver. 1.0, and ROC ver. 1.74.0. A total of three .R files are provided in the supplementary materials. The file “rcode_figs1-3.R” contains the scripts for obtaining Figs 1-3. The file “rcode_fig4.R” contains the scripts to obtain the raw AUC values for one of the scenarios in Fig. 4. The file “functions.R” contains the functions for executing the six methods (edgeR, DESeq2, TCC, and MBCdeg1-3). The third file is required to run the second file. Running the second file with the default parameter settings (i.e., 20 trials, *n*_1_ = *n*_2_ = 3, *P_DEG_* = 0.05, *P*_1_ = 0.5, *FC* = 4, *K* = 3, and *G* = 10,000) produces a tab-delimited file (named “Fig4_0.05_0.5.txt”) containing the raw AUC values of the six methods for individual trials under *P_DEG_* = 0.05 and *P*_1_ = 0.5 in Fig. 4. Image, application 1

Image, application 2

Image, application 3
